# Digital droplet PCR (ddPCR) for the detection and quantification of HPV 16, 18, 33 and 45 - a short report

**DOI:** 10.1007/s13402-017-0331-y

**Published:** 2017-07-26

**Authors:** Gabriella Lillsunde Larsson, Gisela Helenius

**Affiliations:** Department of Laboratory Medicine, Faculty of Medicine and Health, Örebro University, Örebro, Sweden

**Keywords:** Human papilloma virus (HPV), Quantitation, Viral load, PCR

## Abstract

**Purpose:**

Human papilloma virus (HPV) infection is associated with several anogenital malignancies. Here, we set out to evaluate digital droplet PCR (ddPCR) as a tool for HPV 16, 18, 33 and 45 viral load quantification and, in addition, to compare the efficacy of the ddPCR assay for HPV 16 detection with that of quantitative real-time PCR (qPCR).

**Methods:**

Clinical samples, positive for HPV genotypes 16, 18, 33 and 45 were analyzed for viral load using ddPCR. Sample DNA was cleaved before droplet generation and PCR. Droplets positive for VIC and FAM fluorescence were read in a QX200 Droplet reader™ (BIO-RAD) after which the viral load was calculated using Quantasoft software.

**Results:**

We found that DNAs extracted from formalin fixed paraffin embedded (FFPE) tissue samples yielded lower amplification signals compared to those obtained from liquid based cytology (LBC) samples, but they were clearly distinguishable from negative background signals. The viral limit of detection was 1.6 copies of HPV 16, 2.8 copies of HPV 18, 4.6 copies of HPV 33 and 1.6 copies of HPV 45. The mean inter-assay coefficients of variability (CV) for the assays ranged from 3.4 to 7.0%, and the mean intra-assay CV from 2.6 to 8.2%. The viral load in the different cohorts of tumor samples ranged from 154 to 340,200 copies for HPV 16, 244 to 31,300 copies for HPV 18 and 738 to 69,100 copies for HPV 33. One sample positive for HPV 45 contained 1331 viral copies. When comparing qPCR data with ddPCR copy number data, the qPCR values were found to be 1 to 31 times higher.

**Conclusions:**

Separation of fragments in nanodroplets may facilitate the amplification of fragmented human and viral DNA. The method of digital droplet PCR may, thus, provide a new and promising tool for evaluating the HPV viral load in clinical samples.

## Introduction

Human papilloma virus (HPV) infection is the cause of cervical cancer and the virus has also been found to be strongly associated with other anogenital malignancies. For lesion development, a long-term persistent viral infection is required. The oncogene products E6 and E7 from high risk HPV genotypes can affect human cells in several ways, which may ultimately lead to malignant transformation [1] . Currently, many countries, among which Sweden, are changing their cervical screening policy for women 30 years and older, based on data showing that primary HPV screens exhibit a higher sensitivity for detecting CIN2 and CIN 3 lesions compared to cytology alone [2]. Since most HPV infections disappear without ever leading to cancer, triage testing of HPV positive women is needed to avoid overtreatment. While cytology is the preferred triage method, a high HPV viral load, in serial measurements, has been suggested to serve as a predictor for cervical lesion initiation and progression [3–6]. It has been proposed that a viral load measurement may discern a malignant infection from a productive infection [5, 7, 8]. Where HPV is kept as a viral particle, i.e., virion, the viral load is changing dramatically, i.e., either increasing or decreasing rapidly. In contrast, latent infections show slowly increasing loads. In transforming infections the viral load increases at a medium pace, i.e., at doubling or halving speed [6, 7]. Apart from screening purposes, viral load estimations may also be useful for the follow-up of treated women and for epidemiological studies. For these latter purposes, estimations of viral loads in formalin fixed paraffin embedded (FFPE) material are a prerequisite.

Quantitative real-time PCR (qPCR) has for long been the method of choice for viral load estimation based on its broad range of detection and multiplexing capacity. However, for quantification, standard curves are necessary and the efficiency of the method may vary between runs and reactions [9]. Digital PCR assays were first described in 1992 [10] and its applications have included both human and viral gene targets. The method is based on dilution and partitioning of the sample in many reaction chambers or droplets. Using the same setting as for real-time PCR with PCR primers and probes for fluorescence detection, absolute quantities of PCR amplified fragments can be measured from volumetrically defined water-in-oil droplet partitions, i.e., in digital droplet PCR (ddPCR) [9]. Using Poisson’s distribution, the absolute concentration can be calculated without standard curves with an increased precision for target detection [11]. The aim of the current study was to evaluate ddPCR for viral load quantification of the HPV high risk genotypes 16, 18, 33 and 45. In addition, the ddPCR assay data for HPV 16 quantification were compared to qPCR data.

## Materials and methods

### Patient samples and cell lines

Anonymized HPV-positive patient samples obtained from the Örebro University Hospital during the period 2013 to 2016 were used for method optimization. Samples were selected based on HPV 16, 18, 33 or 45 positivity and chosen to replicate both high and low viral load samples as well as representing cervical liquid based cytology (LBC) samples (Preserv Cyt, Hologic Bedford USA) and formalin fixed paraffin embedded (FFPE) samples. The viral load assays for each genotype were tested for specificity using (a) a pooled multi-positive control sample consisting of the high risk HPV genotypes 16, 18, 31, 33, 35, 39, 45, 51, 52, 56, 58 and 59, (b) a pooled multi-positive control sample lacking the sought genotype in a mix of the other high risk genotypes mentioned above and (c) a human HPV-negative sample. The sensitivity of each assay was calculated as the detection of a minimal viral load: limit of detection (LOD). The cell lines CaSKi (ATCC CRL-1550) and SiHA (ATCC HTB-35) were used as controls for HPV 16. The SiHA cell line has been reported to carry 1–2 integrated copies per cell [12] while the CaSKi cell line carries approximately 600 copies per cell [13]. A cohort of primary FFPE tissue samples used consisted of HPV-positive samples from a consecutive vaginal carcinoma series (1975–2002; *n* = 17), as well as from a consecutive vulvar series (1983–2008; *n* = 25), previously described by Lillsunde et al. [14, 15]. The studies were approved by the Ethical committee of the Uppsala-Örebro region, Sweden (Dnr 2008/294).

### HPV genotyping

The samples used for ddPCR optimization were previously HPV tested using Anyplex™ II HPV28 (Seegene, Seoul, Korea) [16]. Briefly, the samples were analyzed for the presence of 28 different genotypes (HPV 6, 11, 16, 18, 26, 31, 33, 35, 39, 42, 43, 44, 45, 51, 52, 53, 54, 56, 58, 59, 60, 61, 66, 68, 69, 70, 73 and 82) in two reactions with semi-quantitative detection after 30, 40 and 50 PCR cycles. The human beta-globulin gene (*HBB*) was used as a control. Samples positive for HPV 16, 18, 33 and 45 were retrieved after genotyping and DNA extracted from the samples was used for ddPCR optimization. The FFPE samples used were previously HPV tested using an in-house qPCR protocol [14, 15]. The samples were analyzed for HPV genotypes 6, 11, 16, 18, 31, 33, 35, 39, 45, 51, 52, 56, 58 and 59. Human control genes were included in order to verify cell content and PCR performance, i.e., for the vulvar carcinoma samples the hydroxymethylbilane synthase (*HMBS*) gene and for the vaginal samples the *HBB* gene. Vaginal and vulvar samples positive for HPV 16 (*n* = 33), HPV 18 (*n* = 4), HPV 33 (*n* = 4) and HPV 45 (*n* = 1) were retrieved after genotyping, after which DNA was extracted for ddPCR.

### qPCR for viral load testing of HPV 16 positive samples

From the FFPE samples, vaginal and vulvar samples positive for HPV 16 were previously quantified for viral load using qPCR as described elsewhere [17]. In short, detection of the viral E6 gene for HPV 16 was performed using qPCR for which standard curves of plasmid pBR322, containing the total HPV 16 genome in a background of human DNA, was used in serial dilutions. A calibration curve of the plasmid dilutions (log_10_) was plotted against the PCR cycles and used for sample copy estimation.

### Digital droplet PCR (ddPCR)

DNA (100 ng) was cleaved using BamH1 (Sigma-Aldrich, Schnelldorf, Germany). The selection of this restriction enzyme was done using ddPCR Calculations Tools, version 10 (BIO-RAD, Hercules, CA, USA). The Mastermix for ddPCR included 1× ddPCR Supermix for Probes (no dUTP, BIO-RAD), 0.9 μM primer and 0.25 μM probe (Applied Biosystems, Hilden, Germany) together with 5 μl cleaved sample DNA. The PCR designs were in duplex, combining each HPV genotype (16, 18, 33 and 45) with the human control *HBB* gene [16]. In addition, new primers and a probe for the HPV 33 assay were developed; forward primer: HPV 33 F: ATATTTCGGGTCGTTGGGCA, reverse primer HPV 33 R: ACGTCACAGTGCAGTTTCTCTACGT and probe: GGACCTCCAACACGCCGCACA. A black hole quencher was used in combination with Fam and VIC fluorescent dye reporters. The Mastermix and sample DNA were thoroughly mixed and transferred to a DG8 Cartridge for a QX100™/QX200 Droplet Generator (BIO-RAD). Next, Droplet Generation Oil for Probes (BIO-RAD) was added to the cartridge which was placed into the QX200 Droplet Generator™ (BIO-RAD). After droplet generation, the droplets were carefully transferred to a twin-tec semi-skirted 96-well PCR plate (Eppendorf AG, Hamburg, Germany) after which the plate was sealed 2 times 4 s at 170 °C using an Axygen Platemax semi-automated plate sealer (Thermo Fisher, Waltham, MA, USA). Subsequent amplification was performed in a Veriti 96-well thermal cycler (Applied Biosystems) with a ramp rate of 2 °C/s. First, the enzyme was activated at 95 °C for 10 min followed by 40 cycles of denaturation at 94 °C for 30 s and 62 °C for one minute. The enzyme was deactivated at 98 °C (10 min) and the reaction was kept at 4 °C. Droplets were read in a QX200 Droplet reader™ (BIO-RAD) after which the ddPCR data were analyzed using Quantasoft Version 1.6.6. Manual thresholds were applied to both the HPV genotype and the human control gene. In each run a HPV-negative human sample and a non-template control were included.

The ddPCR assay was found to have an excellent intra- and inter-assay coefficient of variability (CV) and the assay was successful on both cytology and FFPE material. We found that for the FFPE samples the amplitudes of the positive droplets were lower compared to those of the cytology samples, but they were easily delineated from the negative background. One of the major advantages of ddPCR is amplification in each individual droplet and subsequent endpoint reading [9]. The separation of DNA fragments in compartments circumvents competition between fragments and facilitates the amplification of rare low-copy fragments as well as of fragmented DNA, which is often encountered in FFPE material. The estimation in the HPV 16 positive cell line SiHA of 2 viral copies per cell corresponds well to previous reports [12], whereas in the CaSKi cell line we found almost double the amount of 600 viral copies per cell reported earlier [13]. This latter discrepancy may be due to the DNA hybridization technique used. Alternatively, the CaSKi cell line is known to have concatameric integrations and, as a consequence, some sequences may have been deleted. The E6 gene is often intact upon integration and, therefore, a good choice when designing HPV PCR assays, as was done in the current study.

### Statistics

Droplet reader software results were represented as copies/μl for each target (HPV genotype and control gene). Viral copy numbers/cell were calculated as: (Viral copies/(*HBB* copies/2)). Intra- and inter-assay coefficients of variability (CV) were calculated for method optimization. For descriptive statistics, IBM SPSS Statistics version 22 was used (IBM, New York, NY, USA). The Kruskal-Wallis test was used to compare medians (viral copy and viral copies per cell) between HPV genotypes. For HPV 16, viral load data from Lillsunde et al. [17] were compared to ddPCR viral load data. 20 ng DNA was used in every reaction. The two methods were compared using a Pearson correlation test. To further investigate differences of viral copy numbers between the two methods we used the Wilcoxon sign rank test.

## Results and discussion

**Fig. 1:**
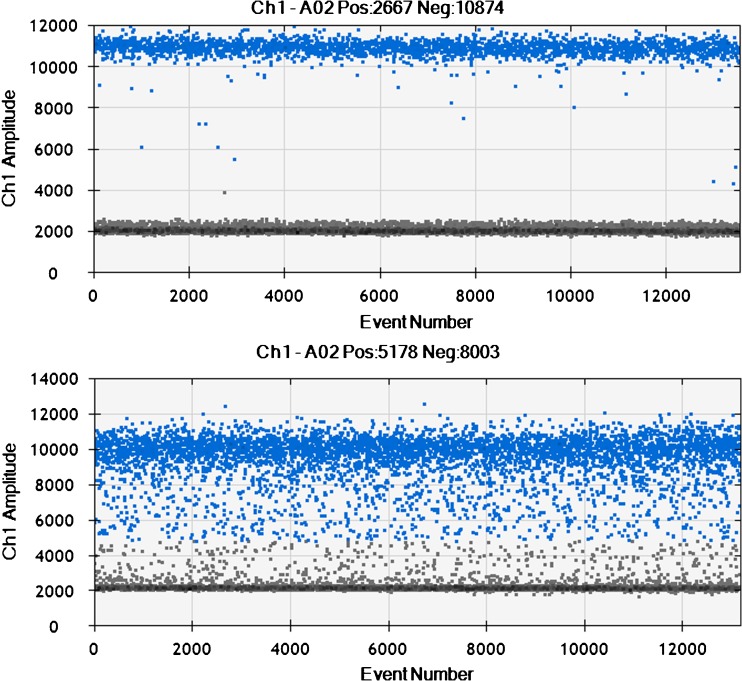
Digital droplet PCR (ddPCR) for HPV 16 detection. Blue droplets = droplets positive for HPV 16, grey droplets = droplets negative for HPV 16. The upper figure shows a HPV 16-positive LBC sample, the lower figure a HPV 16-positive FFPE sample where droplets generally have a lower droplet amplitude

We found that all genotype assays yielded positive droplets with channel amplitude signals between 6000 and 10,000. Manual thresholds for separating negative from positive droplets were applied to the HPV genotypes and the human control gene (*HBB*). We also found that DNA derived from the FFPE samples yielded lower amplification signals compared to the LBC samples (Fig. 1). To determine the sensitivity of each assay, i.e., the viral limit of detection (LOD) that could be repeated, positive samples from each genotype were diluted and analyzed in duplicate. By doing so, we found that the viral LOD in a 20 μl reaction mixture was 1.6 copies of HPV 16, 2.8 copies of HPV 18, 4.6 copies of HPV 33 and 1.6 copies of HPV 45. Five samples positive for HPV 16, 18, 33 and 45 were used to calculate the inter- and intra-assay coefficients of variability (CV). Triplicate results were used for intra-assay and inter-assay calculations. Three different runs were compared. By doing so, we found that HPV 18 had the lowest inter-assay CV (1.2–4.8%) as well as the lowest intra-assay CV (0.2–4.4%) of the four assays (Table 1).

**Table 1:** Inter- and intra-assay coefficient of variability (CV) and viral limit of detection (LOD) for HPV genotyping assays. Five different sample results were used for calculations of inter- and intra-CVs of HPV genotypes 16, 18, 33 and 45 and both range and mean values of variability are presented

	Inter-assay CV (%)	Mean inter-assay CV (%)	Inter-assay CV (%)	Mean intra-assay CV (%)	Viral limit of detection (LOD) in 20 μl reaction
HPV 16	2.4–8.7	5.6	1.3–7.5	4.3	1.6
HPV 18	1.2–4.8	3.4	0.2–4.4	2.6	2.8
HPV 33	0.3–14.6	7.0	2.4–12.2	8.2	4.6
HPV 45	0.1–6.6	3.5	0.7–6.5	3.8	1.6

The primers and probes used for the ddPCR assay have previously been used for qPCR-based HPV detection and genotyping of FFPE samples [16] making them ideal to transfer to ddPCR. Some minor modifications were made (FAM/VIC), since the reactions were performed in duplex, combining each HPV genotype (16, 18, 33 and 45) with the human control gene (*HBB*). This combination allows for copy number per cell estimation and could also provide information on sample adequacy and, possibly, also on the state of infection. Previously, a viral load measurement has been proposed to discriminate a persistent infection from a productive infection [6, 7] and ddPCR could thus be used for a cervical screening triage. However, in order to follow viral replication and disease progression, multiple samples need to be analyzed over a period of time. Viral load has been shown to vary substantially in established HPV-induced carcinomas, and a low viral copy number may reflect integration in the human genome [17]. Also, the concept of latency of HPV in the basal epithelial cells argues for a sensitive detection method [18] that is also compatible with FFPE material [18].

To assess the assay specificity, a multi-positive control cohort including HPV high risk genotypes 16, 18, 31, 33, 35, 39, 45, 51, 52, 56, 58 and 59 was analyzed with each mix (16, 18, 33 and 45) and, by doing so, found to be positive for the expected genotypes. Also, controls were prepared that were negative for the 4 specific genotypes (and positive for the remaining 11 genotypes) and found to be negative. In addition, we found that the SiHA cell line harbored 2 HPV 16 copies/cell and that the CaSKi cell line harbored 1084 HPV 16 copies/cell.

**Fig. 2:**
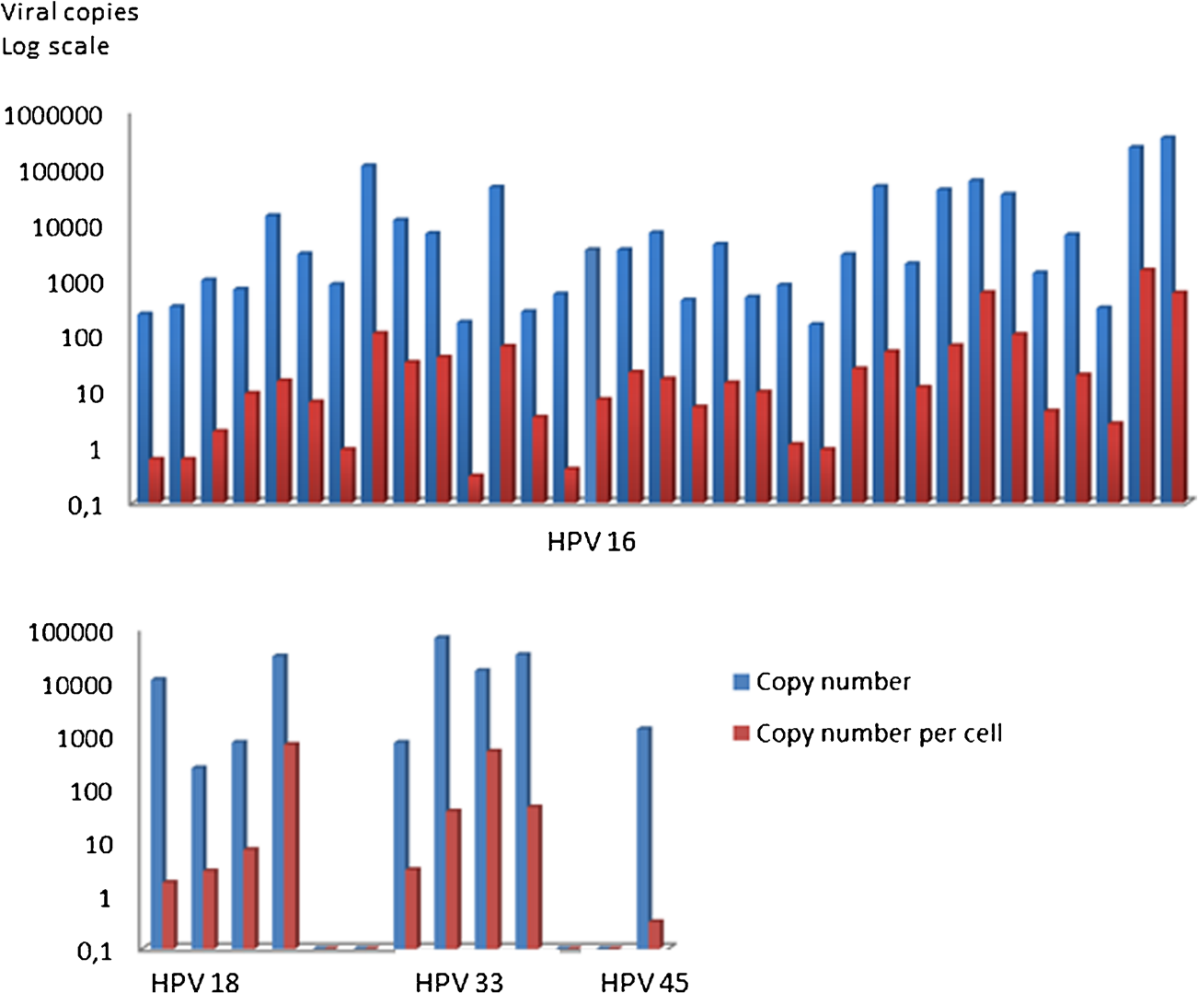
Viral copies and viral copies/cell for HPV 16, 18, 33 and 45 (logarithmic scale). Upper figure: 33 FFPE samples positive for HPV 16 (mean copy number: 29,425, mean viral load/cell: 97). Lower figure: 4 FFPE samples positive for HPV 18 (mean copy number: 10,895, mean viral load/cell: 173) and 4 FFPE samples positive for HPV 33 (mean copy number: 29,994, mean viral load/cell: 148). The median viral copy number (Kruskal-Wallis: *p* = 0.566) or median viral copy number per cell (Kruskal-Wallis: *p* = 0.367) did not differ between the genotypes. HPV 45: single case

Next, a cohort of FFPE study samples (HPV 16; *n* = 33, HPV 18; *n* = 4, HPV 33; *n* = 4 and HPV 45; *n* = 1) was analyzed in duplicate and mean values were used for result evaluation. Two samples were diluted ten-fold to retain negative droplets, which is needed for concentration estimations using the assay software tool. Single results were used and concentrations adjusted to 10-fold. We found that the viral load in 20 ng input total DNA ranged from 154 to 340,200 copies for HPV 16, from 244 to 31,300 copies for HPV 18 and from 738 and 69,100 copies for HPV 33. One sample positive for HPV 45 had 1331 viral copies in 20 ng input DNA. The mean viral copy number for the HPV 16 positive cases (*n* = 33) was 29,425 with a median value of 2820 (SD: 71,284). For the HPV 18 positive cases (*n* = 4) the mean value was 10,895 with a median value of 6019 (SD: 14,525). Finally, the HPV 33 positive cases (*n* = 4) had a mean value of viral copies of 29,994, with a median of 25,070 (SD: 29,290). The median viral copy number did not differ between the genotypes (Kruskal-Wallis: *p* = 0.566). The combination of HPV genotype detection with the human control gene *HBB* allows for an estimation of the number of viral copies per cell. By doing so, we found that the number of HPV copies/cell varied between 0.3 and 1440 in the samples positive for HPV 16, between 1.8 and 680 for HPV 18, between 3 and 506 for HPV 33 and, finally, 0.3 viral copies per cell for the HPV 45 positive sample. The mean viral copy number per cell for the HPV 16 positive cases (*n* = 33) was 97, with a median of 12 (SD: 276). For the HPV 18 positive cases (*n* = 4) the mean viral copy number per cell was 173, with a median of 5 (SD: 338). Finally, the HPV 33 positive cases (*n* = 4) had a mean viral copy number per cell of 148, with a median of 41 (SD: 239). The median viral copy number per cell did not differ between the genotypes (Kruskal-Wallis: *p* = 0.367; Fig. 2).

Hence, we found that the results varied most within the group of HPV 16 positive tumors. This is also the largest group of tumors and clearly shows a diverse constitution. Recently, two studies were published on the efficacy of ddPCR in HPV 16 mRNA level estimations in oropharyngeal cancers. They found that the technique was suitable for assessing the HPV 16 viral load in different sample types with a high sensitivity and specificity compared to assessment based on the commonly used immunohistochemistry (IHC) p16 marker [19, 20]. To the best of our knowledge this is the first report on the use of ddPCR for HPV 18, 33 and 45 detection. The assay may also be used for mRNA assessment, at least in freshly obtained (cytology) samples.

**Fig. 3:**
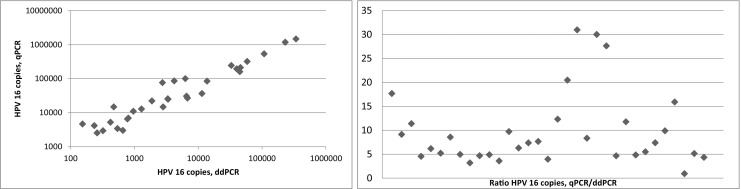
Left: Correlation between ddPCR and qPCR for HPV 16 viral copy load. A strong positive linear relationship was found (Spearman correlation coefficient 0.99). Right: Ratio distribution (qPCR copy number/ddPCR copy number) of 33 HPV 16 positive samples. Mean of samples: 9.7 (SD: 7.7)

Subsequently, the viral copy results obtained by ddPCR and qPCR were compared in 33 HPV 16 positive vaginal and vulvar FFPE tumor samples. By using correlation analysis, a strong association was found (Spearman correlation coefficient 0.99, *p* = 0.000). In all but one individual sample, the ddPCR copy number results were lower compared to the copy number results obtained by qPCR (Wilcoxon sign rank test: *p* = 0.000). Compared to ddPCR, the copy numbers obtained by qPCR were between 1 and 31 times higher (mean: 9.7; SD 7.7) (Fig. 3). Despite the fact that the viral loads per sample followed similar patterns, the ratios between the methods (qPCR copy number/ddPCR copy number) varied considerably. This observation may at least partially be explained by differences in assay construction, i.e., different primer/probes were used, although both were designed to detect the E6 gene. A more plausible explanation may, however, be matrix related. In the qPCR setting, the tumor samples were compared to a standard curve of a plasmid construct for which 10-fold dilution steps were used. A theoretical nucleotide mass was used for calculation and subsequent dilution. When analyzing the plasmid dilutions using the ddPCR HPV 16 assay (data not shown), the dilutions turned out to be 3 to 4 times lower than theoretically expected. Thus, miscalculation, measurement deviation or technical (pipetting) errors may be explanatory variables underlying this latter result. The impact of all mentioned variables on the end result, apart from the additional workload, is a major drawback of using dilution curves and argues in favor of using ddPCR.

From our data we conclude that the separation of fragments in nanodroplets may facilitate the amplification of fragmented human and viral DNA and that the ddPCR method is suitable both for LBC samples and archival FFPE samples. The ddPCR method thus represents a new promising tool for evaluating the HPV viral load.
